# Comprehensive knowledge about HIV/AIDS and associated factors among reproductive age women in Liberia

**DOI:** 10.1186/s12889-024-18105-9

**Published:** 2024-02-26

**Authors:** Beminate Lemma Seifu, Gilbert Eshun, Getayeneh Antehunegn Tesema, Frank Kyei-Arthur

**Affiliations:** 1 Department of Public Health, College of Medicine and Health Sciences, Samara University, Samara, Ethiopia; 2 Seventh Day Adventist Hospital, Agona-Asamang, Ghana; 3 Department of Epidemiology and Biostatistics, Institute of Public Health, College of Medicine and Health Sciences, and comprehensive specialized Hospital, University of Gondar, Gondar, Ethiopia; 4 Department of Environment and Public Health, University of Environment and Sustainable Development, Somanya, Ghana

**Keywords:** Comprehensive knowledge about HIV/AIDS, Liberia, Demographic and Health Survey, HIV/AIDS, Reproductive age women

## Abstract

**Introduction:**

Acquired Immune Deficiency Syndrome (AIDS) continues to be a deadly pandemic and a serious threat to public health. Globally, reproductive age women are more likely to be infected with Human Immunodeficiency Virus (HIV). Comprehensive knowledge about HIV/AIDS is pivotal in the fight against AIDS. However, comprehensive HIV/AIDS knowledge is low in Sub-Saharan African (SSA) nations including Liberia, which contributes to the high incidence of HIV in these nations. This study assessed the level of comprehensive knowledge about HIV/AIDS and its associated factors among reproductive age women in Liberia.

**Methods:**

The prevalence and associated factors of comprehensive knowledge about HIV/AIDS among reproductive age women in Liberia were determined using secondary data analysis of 2019–2020 Liberia Demographic and Health Surveys (LDHS). Comprehensive knowledge about HIV/AIDS was a composite variable computed from six variables available in the Demographic and Health Survey (DHS). The study included 7,621 reproductive age women in weighted samples. A generalized linear mixed model with robust error variance was used. For the variables included in the final model, adjusted prevalence ratios (aPR) with 95% confidence intervals (CI) were calculated.

**Results:**

The prevalence of comprehensive HIV/AIDS knowledge among Liberian women aged 15–49 was 33.5%. Women’s age and education, and distance to health facility were positively associated with comprehensive knowledge about HIV/AIDS among Liberian reproductive age women. In contrast, community poverty level was negatively associated with comprehensive knowledge about HIV/AIDS.

**Conclusion and recommendations:**

This study demonstrates that the prevalence of good comprehensive HIV/AIDS knowledge was relatively low among reproductive age women in Liberia. Hence, health practitioners and policymakers should strengthen HIV/AIDS sensitization programmes to increase women’s knowledge about HIV/AIDS.

## Introduction

Acquired Immune Deficiency Syndrome (AIDS) caused by the Human Immunodeficiency Virus (HIV) continues to be a deadly pandemic, which also happens to be a serious threat to public health [1, 2]. Globally, it has been estimated that the virus has infected approximately 76 million people since the beginning of the infection in 1981 [3]. An estimated 38.4 million people are living with the infection, of which two-thirds occurred in sub-Sahara Africa (SSA) and 1.5 million people acquired the disease in 2021 [1, 4]. The number of deaths from HIV/AIDS and its related illness is staggering, as it has claimed the lives of 40.1 million people so far with 650,000 deaths in 2021 [1, 4]. In Liberia, there were 1,100 deaths related to HIV/AIDS in 2021 while 35,000 children aged between 0 and 17 have been made orphans because of the disease in the country [5].

Various organized institutions and governments in the fight against HIV/AIDS with the sole purpose of ending the pandemic had made investments, efforts and strategic plans [4, 6]. The United Nations (UN) in the quest to accelerate the progress towards combating HIV/AIDS set to achieve 95-95-95 target by 2025, which is spearheaded by the United Nations Program on HIV/AIDS (UNAIDS). This aims for at least 95% of PLWHA to know their status, of whom at least 95% should be on treatment and out of which at least 95% should have achieved viral suppression by 2030 [ 1, 4, 8]. In 2022, the percentage of people living with HIV who know their status in Liberia was 74%, with 72% of them been on antiretroviral therapy, and those of them with suppressed viral loads were unknown [5]. Also, the United Nations Sustainable Development Goal 3 (good health and well-being for all) aims to end the epidemics of AIDS by 2030, but the high new infections recorded yearly and the disruption of HIV services due to the COVID-19 pandemic is an impediment to attaining this [1, 6].

The occurrence of high new infection can be partly attributed to low comprehensive knowledge about HIV/AIDS and the available prevention methods [7]. Comprehensive knowledge is having or exhibiting a complete, thorough and wide mental grasp over a subject such as HIV/AIDS [8]. In this paper, a comprehensive knowledge about human immunodeficiency virus (HIV) includes a thorough understanding of route of HIV transmission and not holding on to local misconceptions about infection. Comprehensive knowledge has been found to be a predictor of health attitudes towards infections since knowledge influences attitude [8]. It also helps in correcting misconceptions and myths about a disease, as well as increasing the perception of susceptibility of an individual to an infection [8]. Though many countries in SSA have achieved the second and third of the 95–95–95 targets for 2025, none has achieved the first target of over 95% of PLWHA knowing their HIV status [6]. Comprehensive knowledge about HIV/AIDS in SSA have been found to be low with 19.3% and 48.9% in Ethiopia and Burundi, respectively [9], 51.9% in Uganda [10] and 59% in Ghana [11]. Comprehensive knowledge about HIV/AIDS is important and pivotal in the fight against the pandemic. It enables individuals to acquire the correct knowledge and perspective on HIV/AIDS, its transmission and methods for prevention thereby reducing the incidence [9, 12]. It facilitates the need for awareness of testing and knowing HIV status [13, 14], enable early treatment, care and support of PLWHA [9]. Studies have identified age, education, wealth status, residence, sex of the family’s head, and media exposure as some of the factors that are associated with comprehensive knowledge about HIV [7–11, 15]. In addition, good socioeconomic status is significantly related to comprehensive knowledge on HIV/AIDS [16].

Estimates in 2017 by UNAIDS shows that globally, young women (aged 15-24years) are twice at risk of the infection as compared to their male counterparts in the same age group [17]. This is due to biological factors (i.e. being more exposed to bodily fluids during sex), gender inequality and discrimination, inadequate sexual autonomy and decision-making power, intimate partner violence, poor access to sexual and reproductive health, limited education and economic opportunities [17, 18]. At the end of 2021, 54% of all people living with HIV/AIDS (PLWHA) globally are women and girls [4]. In SSA, this made profound as 63% of all new HIV infections in 2021 were recorded among women and girls [4]. Likewise, in Liberia the prevalence of HIV among reproductive age women (1.4%) is higher than men (0.8) which is statistically significant [5]. In addition, in the same country, the prevalence of HIV in young women is twice that in young men [5]. In Liberia there are 32, 000 adults aged 15 and above who are living with HIV/AIDS, of which 20, 000 are women [5].

Though women of reproductive age in Sub-Sahara Africa are at higher risk of acquiring the infection [17], comprehensive knowledge about HIV/AIDS among this group is low (38.56%) in the region according to a multilevel analysis of DHS data conducted in 2022 [7]. Currently, to the best of our knowledge, there have been no published study on comprehensive knowledge about HIV/AIDS and its associated factors among reproductive age women in Liberia. In light of this, this study assesses the level of comprehensive knowledge about HIV/AIDS and its associated factors among women aged 15–49 in Liberia. This study will help stakeholders, policymakers and program planners to adopt measures and strategies that could potentially increase HIV/AIDS awareness in order to end the pandemic.

## Methods

### Data source and sampling procedure

**Fig. 1:**
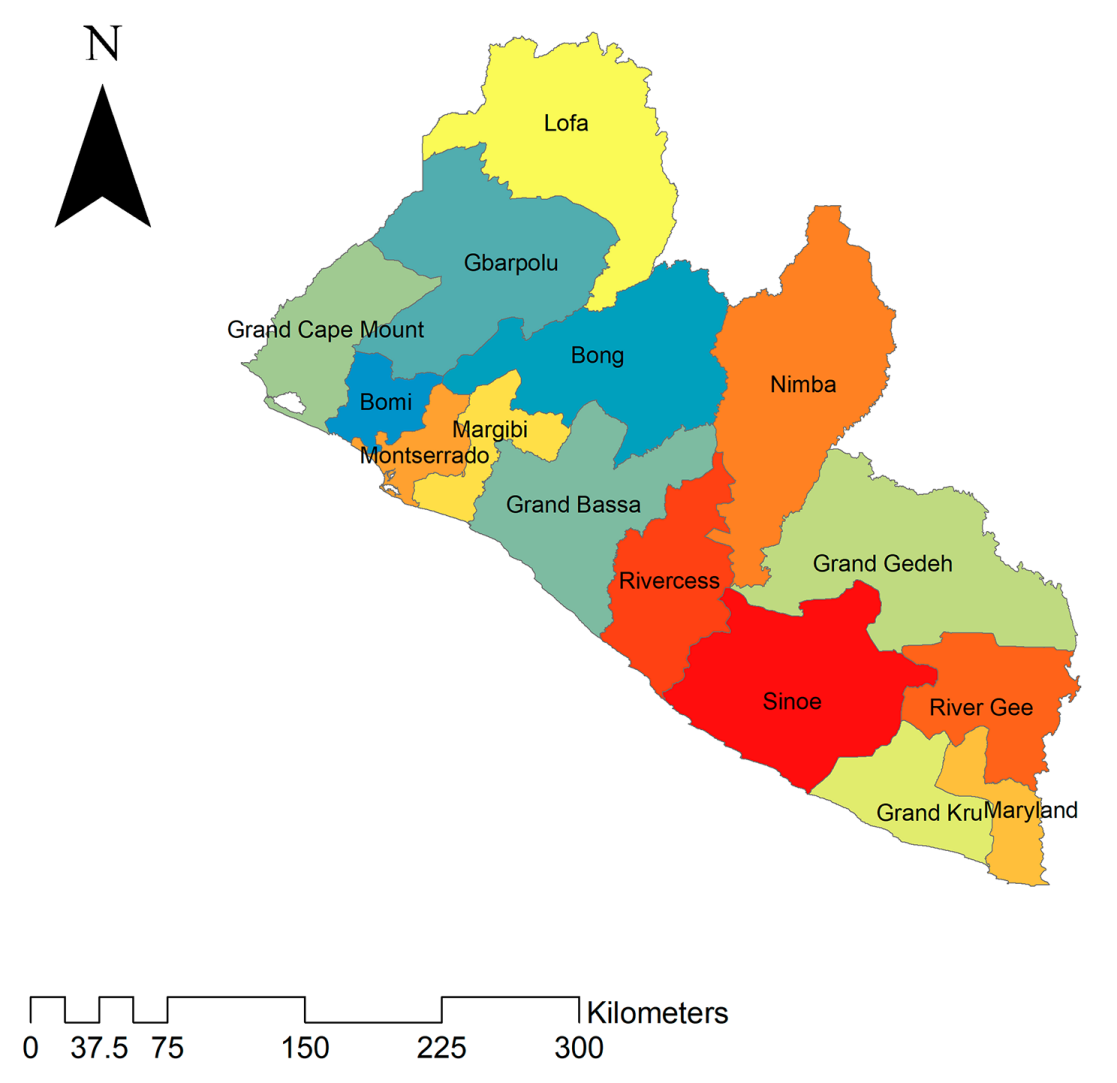
Map of counties in Liberia

A secondary data was analysed based on the 2019-20 Liberian Demographic and Health Survey (LDHS) data. Liberia is located on the west Coast of Sub-Saharan Africa (Fig. 1). It borders Sierra Leone to the northwest, Guinea to the northeast, Ivory Coast to the east, and the North Atlantic Ocean to the southwest. The 2019-20 LDHS is the 5th DHS survey primarily conducted to provide up-to-date estimates of key demographic and health indicators necessary for program managers, policymakers, and implementers to evaluate the impact of existing policies and programs. A multi-stage stratified cluster sampling technique was employed to recruit the samples. The 2008 national and Population Health Census was used as a sampling frame. After selecting the Enumeration Areas (EAs), household listing operation was conducted as the 2008 PHC has passed 15 years. Liberia’s 15 counties are grouped to form five geographical regions, with each region consisting of three counties. Each county is divided into districts and each district is into clans. Then each clan was further subdivided into EAs. The primary and secondary sampling units were EAs and households, respectively. A total of 325 EAs and on average 30 households in each selected EAs were considered. The survey consists of different datasets including men, women, children, birth, and household datasets. For this study, we used the Individual Record dataset (IR file). A total weighted sample of 7,621 reproductive-age women was considered for the final analysis.

### Study variables and measurements

#### Dependent variable

Comprehensive knowledge about HIV/AIDS was the dependent variable. It is a latent variable, that can be generated by computing six variables available in DHS. It was a composite score of the following six questions (each has two options “no” and “yes”; “can reduce risk of getting HIV/AIDS through using of condoms during sex?”, “can reduce risk of getting HIV by having one sex partner only”, “a healthy-looking person can have HIV?“, “can get HIV by witchcraft or supernatural means?“, “can get HIV from a mosquito bite?” and can get HIV by sharing food with a person who has HIV/AIDS?“). Comprehensive knowledge about HIV/AIDS was defined when a woman correctly answered all the six questions, that is “yes” for the first three questions and “no” for the remaining three questions [19, 20].

### Independent variables

Considering the hierarchical nature of DHS data where women are nested within the cluster, two levels of independent variables were considered at two levels. Age of respondent, marital status, woman’s educational status, wealth status, reading newspapers, listening to the radio, watching television, women’s health care decision-making autonomy, covered by health insurance, husband’s educational status, religion, and woman’s occupational status were the individual level independent variables. Whereas, residence, distance to the health facility, region, community women’s education, community media exposure, and community poverty were considered the community-level independent variables considered in this study.

#### Community literacy level

The proportion of women in the cluster who attended primary, secondary, and higher education. The sum of individual women’s primary, secondary, and higher educational attainment might reveal the cluster’s overall academic stand. The group was divided based on national median value, with one category having more educated women and the other having fewer.

#### Community poverty level

The percentage of poor and poorest mothers in the cluster is specified. The proportion of poor and poorest women in each cluster was aggregated to reflect the overall poverty status within the cluster. Mothers were categorized based on their poverty level compared to the national median value.

**Community media exposure**: the proportion of women in a cluster who had media exposure. The media exposure of women in the cluster was calculated by combining the media exposure of individual women. The national median value was used to divide the community media exposure into two categories: higher and lower.

### Data management and analysis

To ensure accurate statistical analysis, we applied weightings to the data based on sampling weight, primary sampling unit, and strata. This was done to restore the survey’s representativeness and account for the sampling design when computing standard errors. The aim was to obtain reliable statistical estimates. To manage and analyze our data, we used STATA version 17 statistical software. Given the study design and prevalence of comprehensive knowledge about HIV/AIDS (> 10%), multilevel modified poisson regression with robust variance was fitted.

We preferred this model because of the following reasons. For start, when the magnitude of the outcome variable is common, the odds ratio obtained using the binary logistic regression approach overestimates the strength of the relationship. Second, because the DHS data is hierarchical, mothers were nested within cluster/EA. As a result, our model considers data dependencies as well as the problem of overestimation.

The likelihood Ratio (LR) test, Intra-class Correlation Coefficient (ICC), were computed to measure the variation between clusters. The ICC quantifies the degree of heterogeneity between clusters.

$ICC= {\delta }^{2}/{(\partial }^{2}+ {\pi }^{2})$ [21], $\sigma$^2^ indicates that cluster variance.

We have fitted four models separately. Model 1 (null model) was fitted without independent variables to estimate the cluster-level variation of the comprehensive knowledge about HIV/AIDS in Liberia. Model 2 and Model 3 were adjusted for individual-level variables and community-level variables, respectively. Model 4 was the final model adjusted for individual and community-level variables simultaneously. Variables with a p-value < 0.2 in the bi-variable multilevel modified Poisson regression analysis were considered for the multivariable analysis. Deviance was used to verify model fitness and a model with the lowest deviance was considered the best-fit model. Finally, the Adjusted Prevalence Ratio (APR) with its 95% confidence interval (CI) was reported, and variables with a p-value < 0.05 in the multivariable analysis.

## Results

### Socio-demographic and economic characteristics of the study participants

A total of 7,621 reproductive age women were included in this study. About half (52.13%) of the reproductive age women were married, and 29.33% of them do not attend formal education. More than two-fifth (38.50%) of the study participants had no formal employment and the vast majority of the participants (96.05%) had no health insurance coverage. In this study, Christians accounted for about 84.85% and Muslims 13.68%. Nearly two-thirds (62.66%) of women resided in urban households (Table 1).

### Prevalence of comprehensive knowledge about HIV/AIDS by study variables among reproductive age Liberian women

The prevalence of comprehensive HIV/AIDS knowledge among Liberian women was 33.49% (95% CI: 32.45%, 34.55%). Comprehensive knowledge towards HIV/AIDS among women who attained higher, secondary and primary level of education were 65.5%, 42.47% and 23.38%, respectively. The proportion of comprehensive knowledge towards HIV/AIDS among women with health insurance was 46.26% (Table 1).

**Table 1:** Socio-demographic and economic characteristics and the prevalence of comprehensive HIV/AIDS knowledge among reproductive age women in Liberia, 2019–2020 (*n* = 7,621)

Variable	Total weighted frequency (%)	Comprehensive HIV/AIDS knowledge		p-value
		No (%)	Yes (%)	
**comprehensive HIV/AIDS knowledge**		**5,069 (66.51)**	**2,552 (33.49)**	
**Individual level variables**				
**Age**				
15–24	2,932 (38.47)	1,986 (67.74)	946 (32.26)	< 0.001
25–34	2,375 (31.16)	1,451 (61.09)	924 (38.91)	
35–49	2,314 (30.37)	1,632 (70.51)	682 (29.49)	
**Marital status**				
Not married	2,946 (38.66)	1,849 (62.76)	1,096 (37.21)	0.533
Married/ in union	3,973 (52.13)	2,743 (69.05)	1,230 (30.95)	
Widowed/ divorced/separated	702 (9.22)	477 (67.84)	226 (32.16)	
**Woman’s educational status**				
No formal education	2,236 (29.33)	1,739 (77.78)	497 (22.22)	< 0.001
Primary	1,780 (23.36)	1,364 (76.62)	416 (23.38)	
Secondary	3,135 (41.14)	1,803 (57.53)	1,332 (42.47)	
Higher	470 (6.16)	162 (34.46)	308 (65.54)	
**Husband’s educational status**				
No formal education	993 (26.52)	781 (78.67)	212 (21.33)	< 0.001
Primary	482 (12.88)	367 (76.10)	115 (23.90)	
Secondary	1,788 (47.76)	1,197 (66.93)	591 (33.07)	
Higher	481 (12.85)	226 (46.92)	255 (53.08)	
**Woman’s employment**				
Not employed	2,935 (38.50)	1,839 (62.65)	1,096 (37.35)	0.001
Employed	4,687 (61.50)	3,229 (68.91)	1,457 (31.09)	
**Religion**				
Christian	6,467 (84.85)	4,268 (65.99)	2,199 (34.01)	0.001
Muslim	1,043 (13.68)	706 (67.76)	336 (32.24)	
Other	112 (1.47)	95 (84.77)	17 (15.23)	
**Household wealth status**				
Poorer	1,273 (16.71)	1,003 (78.79)	270 (21.21)	< 0.001
Poor	1,320 (17.32)	983 (74.47)	337 (25.53)	
Middle	1,422 (18.66)	943 (66.28)	480 (33.72)	
Rich	1,764 (23.14)	1,101 (62.49)	661 (37.51)	
Richest	1,843 (24.18)	1,039 (56.39)	804 (43.61)	
**Woman’s decision-making autonomy**				
Not autonomous	3,994 (52.40)	2,579 (64.57)	1,415 (35.43)	0.01
Autonomous	3,628 (47.60)	2,490 (68.61)	1,139 (31.39)	
**Covered by health insurance**				
No	7,321 (96.05)	4,906 (67.02)	2,414 (32.98)	< 0.001
Yes	301 (3.95)	162 (53.74)	139 (46.26)	
**Read newspaper**				
No	6,638 (87.09)	4,512 (67.97)	2,126 (32.03)	< 0.001
Yes	984 (12.91)	557 (56.66)	426 (43.34)	
**Listen to radio**				
No	3,217 (42.21)	2,179 (67.74)	1,037 (32.26)	< 0.001
Yes	4,404 (57.79)	2,890 (65.61)	1,515 (34.39)	
**Watch television**				
No	4,777 (62.67)	3,336 (69.84)	1,441 (30.16)	< 0.001
Yes	2,845 (37.33)	1,732 (60.91)	1,112 (39.10)	
**Community level variable**				
**Residence**				
Urban	4,776 (62.66)	2,984 (62.49)	1,791 (37.51)	< 0.001
Rural	2,846 (37.34)	2,085 (73.25)	761 (26.75)	
**Distance to health facility**				
Big problem	2,112 (27.71)	1,580 (74.85)	531 (25.15)	< 0.001
Not a big problem	5,509 (72.29)	3,489 (63.32)	2,021 (36.68)	
**Community maternal education**				
Low levels of illiteracy	5,290 (69.40)	3,272 (61.86)	2,017 (38.14)	< 0.001
High levels of illiteracy	2,332 (30.60)	1,797 (77.05)	535 (22.95)	
**Community media exposure**				
Low	4,230 (55.49)	2,742 (64.84)	1,487 (35.16)	< 0.001
High	3,392 (44.51)	2,327 (68.61)	1,065 (31.39)	
**Community poverty level**				
Low poverty level	5,420 (71.11)	3,345 (61.71)	2,075 (38.29)	< 0.001
High poverty level	2,202 (28.89)	1,724 (78.33)	477 (21.67)	

*Others: traditional religion, no religion and other

### Factors associated with comprehensive knowledge towards HIV/AIDS

Age, woman’s educational status, distance from the health facility were significantly associated with increased prevalence of comprehensive HIV/AIDS knowledge. Whereas, belonging to community with high poverty was significantly associated with lower prevalence of comprehensive HIV/AIDS knowledge.

Women aged 25–34 and 35–49 had 31% (aPR = 1.31, 95% CI: 1.20, 1.41) and 29% (aPR = 1.29, 95% CI: 1.18, 1.42) increased prevalence of comprehensive knowledge about HIV/AIDS than women aged 15–24, respectively. The prevalence of having comprehensive knowledge towards HIV/AIDS among women who had primary, secondary and higher education were 1.12 times (aPR = 1.12, 95% CI: 1.01, 1.24), 1.80 times (aPR = 1.80, 95% CI:1.62, 2.01) and 2.33 times (aPR = 2.33, 95% CI 2.02, 2.68) times higher than women who had no formal education, respectively. The prevalence of having a comprehensive knowledge about HIV/AIDS among women who perceived distance to health facility as not big problem were by 12% (aPR = 1.12, 95% CI: 1.01, 1.24) higher than women who perceived distance to health facility as a “big problem”. The prevalence of comprehensive knowledge about HIV/AIDS among women belonged to community with high poverty were decreased by 21% (aPR = 0.79, 95% CI: 0.68, 0.93) than women reside in a community with low poverty level (Table 2).

**Table 2:** Factors associated with comprehensive HIV/AIDS knowledge among Liberian reproductive age women, 2019–2020

Variable	Comprehensive HIV/AIDS knowledge	
	uPR	aPR
**Individual level variables**		
**Age (in years)**		
15–24	1	1
25–34	1.24 (1.15, 1.34)	1.31 (1.20, 1.41)^*^
35–49	1.03 (0.95, 1.12)	1.29 (1.18, 1.42)^*^
**Woman’s educational status**		
No formal education	1	1
Primary	1.06 (0.96, 1.17)	1.12 (1.01, 1.24)^*^
Secondary	1.85 (1.68, 2.03)	1.80 (1.62, 2.01)^*^
Higher	2.70 (2.39, 3.05)	2.33 (2.02, 2.68)^*^
**Woman’s employment**		
Not employed	1	1
Employed	0.95 (0.88, 1.02)	0.94 (0.87, 1.03)
**Religion**		
Other	1	1
Christian	1.67 (1.06, 2.61)	1.41 (0.91, 2.19)
Muslim	1.56 (0.98, 2.49)	1.40 (0.89, 2.21)
**Wealth index**		
Poorest	1	
Poorer	1.24 (1.02, 1.40)	1.07 (0.95, 1.20)
Middle	1.49 (1.31, 1.68)	1.09 (0.96, 1.24)
Richer	1.72 (1.51, 1.96)	1.05 (0.89, 1.23)
Richest	1.96 (1.71, 2.25)	1.04 (0.87, 1.23)
**Covered by health insurance**		
No	1	
Yes	1.28 (1.08, 1.52)	1.07 (0.89, 1.27)
**Read newspaper**		
No	1	
Yes	1.38 (1.25, 1.52)	0.98 (0.89, 1.08)
**Listen to radio**		
No	1	
Yes	1.21 (1.12, 1.31)	1.05 (0.97, 1.15)
**Watching television**		
No	1	
Yes	1.26 (1.17, 1.36)	1.05 (0.97, 1.14)
**Community level variables**		
**Residence**		
Urban	1	
Rural	0.67 (0.59, 0.75)	1.08 (0.94, 1.24)
**Distance to health facility**		
Big problem	1	
Not a big problem	1.27 (1.15, 1.41)	1.12 (1.01, 1.24)^*^
**Community education level**		
Low illiteracy level	1	
High illiteracy level	0.63 (0.56, 0.70)	0.91 (0.78, 1.04)
**Community media exposure**		
Low		
High	0.77 (0.68, 0.86)	0.92 (0.82, 1.02)
**Community poverty level**		
Low poverty level	1	
High poverty level	0.59 (0.53, 0.66)	0.79 (0.68, 0.93)^*^

^*^p ≤ 0.05, uPR: unadjusted Prevalence Ratio, aPR: Adjusted Prevalence Ratio

## Discussion

This study aimed to assess the level of comprehensive knowledge about HIV/AIDS and its associated factors among women in their reproductive ages (15–49 years) in Liberia. The prevalence of good comprehensive HIV/AIDS knowledge (33.5%) in this study is higher than the prevalence of comprehensive HIV/AIDS knowledge reported in previous studies in Ethiopia (25.2%) [21] and Iran (19.1%) [22]. The prevalence is apparently similar to that observed in a study carried out in Ghana (31%) [11]. However, the prevalence of comprehensive HIV/AIDS knowledge reported in this study was lower than those reported in Uganda (51.9%) [10], Kenya (54%) [23], and Vietnam (42.4%) [24]. The discrepancies in the prevalence of comprehensive HIV/AIDS knowledge can be partly attributed to differences in the effectiveness of HIV/AIDS educational interventions in these countries. Also, studies in Uganda [10], Ethiopia [21] and Iran [22] were conducted among reproductive aged women, while the study in Ghana [11] was conducted among males and females aged 15–49. Also, the study in Kenya [23] was conducted among women aged 15–24.

Furthermore, there were diversities in the measurement of comprehensive HIV/AIDS knowledge. For instance, Fenny et al.’s [11] study in Ghana measured comprehensive knowledge of HIV using four questions: (1) can a healthy-looking person get HIV? (2) can you get HIV by supernatural means? (3) can you get HIV by sharing food with a person who has HIV/AIDS? and (4) can you get HIV from a mosquito bite?. Also, Ochako et al.’s [23] study in Kenya measured comprehensive knowledge of HIV using five questions: (1) can reduce the risk of getting HIV/AIDS through the use of condoms during sex? (2) can reduce the risk of getting HIV by having one sex partner only? (3) can you get HIV from a mosquito bite? (4) can you get HIV by sharing food with a person who has HIV/AIDS? and (5) can a healthy-looking person get HIV?. Estifanos et al.’s [10] study in Uganda measured comprehensive knowledge of HIV using five questions: (1) can reduce the risk of getting HIV/AIDS through the use of condoms during sex? (2) can reduce the risk of getting HIV by having one sex partner only? (3) can a healthy-looking person get HIV? (4) can you get HIV from a mosquito bite? and (5) can you get HIV by witchcraft or supernatural means?. Study by Teshale et al. [9] measured comprehensive knowledge about HIV/AIDS using the following questions (1) can reduce the risk of getting HIV/AIDS through the use of condoms during sex? (2) can reduce the risk of getting HIV by having one sex partner only? (3) can a healthy-looking person get HIV?. (4) can you get HIV from a mosquito bite? (5) can you get HIV by sharing food with a person who has HIV/AIDS, and (6) can you get HIV by witchcraft or supernatural means?. However, Son et al.’s [24] study in Vietnam measured comprehensive HIV/AIDS knowledge as measured in our study.

The results show that women aged 25–34 and 35–49 were more likely to have a good comprehensive understanding of HIV/AIDS than women aged 15–24. This finding corroborates previous studies, which found that older women have a good comprehensive understanding of HIV/AIDS than younger women [10, 21, 22, 24]. This could be due to older women having higher odds of higher parity than young women do. Thus, during antenatal visits, women are educated and tested for HIV [25, 26], which may explain a good comprehensive understanding of HIV/AIDS among older women than younger women may.

In this study, women with primary, secondary and higher education were more likely to have a good comprehensive understanding of HIV/AIDS than women with no formal education. This finding supports previous studies conducted in Uganda in 2016 [10], Ethiopia in 2016 [21], Iran in 2015 [22], Vietnam in 2000–2014 [24], and Nigeria and Democratic Republic of Congo in 2013/2014 [27] that found that a comprehensive understanding of HIV/AIDS was higher among women with higher education than those with no formal education or primary or less education. Higher education increases women’s exposure to and understanding of HIV/AIDS knowledge [28]. This finding suggests that women without formal education are vulnerable, so health practitioners and policymakers should target them for HIV/AIDS sensitization programmes.

Women who reported that distance to health facilities was not a big problem were more likely to have a good comprehensive understanding of HIV/AIDS than women who indicated distance to health facilities was a big problem. This finding is similar to previous studies in Uganda [29] and Mozambique [30]. Health facilities and personnel are important sources of HIV/AIDS knowledge [31, 32], so easy access to health facilities and personnel may increase women’s exposure to HIV/AIDS knowledge. It is therefore not surprising that women with no challenge with distance to health facilities were more likely to have a good comprehensive understanding of HIV/AIDS than those with challenges with distance to health facilities.

In addition, women whose communities had high poverty levels were less likely to have good comprehensive HIV/AIDS knowledge than those whose communities with low poverty levels. This finding supports previous studies [10, 21, 22, 24] that found that comprehensive understanding of HIV/AIDS was higher among women with higher (richer and richest) wealth quintile than those with low (poorest and poorer) wealth quintile. An explanation is that a higher wealth quintile increases social media exposure and access to varied health information, including HIV/AIDS information [33, 34].

### Strength and limitation of study

The strength of this study was the use of weighted data to ensure representativeness at the national level. Therefore, it can be generalized to all reproductive age women in Liberia during the study period. This study has some limitations. First, this study was cross-sectional, so causality between the dependent and independent variables cannot be established. Second, respondents’ responses were self-reported, and they had to recall their experiences. Therefore, there could be recall bias. Third, although comprehensive knowledge about HIV/AIDS was a latent variable, a generalized linear mixed model was used to measure comprehensive knowledge about HIV/AIDS since we were interested in accounting for correlation among observations within the same cluster. Fourth, we did not use a validated scale for the measurement of comprehensive knowledge about HIV/AIDS. Despite these limitations, this study will contribute to the literature on comprehensive knowledge about HIV/AIDS and its associated factors among reproductive women in Liberia.

## Conclusion

This study demonstrates that the prevalence of good comprehensive HIV/AIDS knowledge was relatively low among reproductive age women in Liberia. Hence, health practitioners and policymakers should strengthen HIV/AIDS sensitization programmes to increase women’s knowledge about HIV/AIDS. The study found that individual-level (women’s age and education) and community-level characteristics (distance to health facility and community poverty level) were significant predictors of comprehensive HIV/AIDS knowledge. Therefore, health practitioners and policymakers should target these characteristics in their development of programmes and interventions to increase women’s comprehensive HIV/AIDS knowledge.

### Strength and limitation of study

The strength of this study was the use of weighted data to ensure representativeness at the national level. Therefore, it can be generalized to all reproductive age women in Liberia during the study period. This study has some limitations. First, this study was cross-sectional, so causality between the dependent and independent variables cannot be established. Second, respondents’ responses were self-reported, and they had to recall their experiences. Therefore, there could be recall bias. Despite these limitations, this study will contribute to the literature on comprehensive knowledge about HIV/AIDS and its associated factors among reproductive women in Liberia.

## Data Availability

All result-based data are in the manuscript. In addition, the dataset can be accessed from the measure DHS Program through https://www.dhsprogram.com.

## Abbreviations

AIDS: Acquired immunodeficiency virus
aPR: Adjusted Prevalence Ratio
CI: Confidence interval. DHS:Demographic and Health Survey
EA: Enumeration Area
HIV: Human immunodeficiency virus
ICC: Intra-class correlation coefficient
LDHS: Liberian Demographic and Health Survey
LR: likelihood Ratio
MOR: Median odds ratio
PLWHA: People living with HIV/AIDS
SSA: Sub-Saharan African
UN: United Nations
UNAIDS: United Nations Program on HIV/AIDS
uPR: Unadjusted Prevalence Ratio
